# Efficacy of electroacupuncture for insomnia in cancer patients: a systematic review and meta-analysis

**DOI:** 10.3389/fneur.2025.1512052

**Published:** 2025-02-10

**Authors:** Xiaodong Liu, Ning Xu, Shangpei Wang, Qingjun Jia

**Affiliations:** ^1^Department of Comprehensive Support, Hangzhou Center for Disease Control and Prevention (Hangzhou Health Supervision Institution), Hangzhou, China; ^2^Department of Radiology, The Second Hospital of Anhui Medical University, Hefei, China; ^3^Department of Tuberculosis Control and Prevention, Hangzhou Center for Disease Control and Prevention (Hangzhou Health Supervision Institution), Hangzhou, China

**Keywords:** electroacupuncture, cancer, insomnia, sleep, meta-analysis

## Abstract

**Background:**

Insomnia is a prevalent symptom among cancer patients. Electroacupuncture (EA) has been widely applied in managing sleep disorders, particularly in cancer patients or those experiencing insomnia.

**Objectives:**

This meta-analysis aims to evaluate the efficacy and safety of electroacupuncture for treating cancer-related insomnia.

**Methods:**

Two independent reviewers conducted comprehensive searches across multiple databases, including EMBASE, Web of Science, PubMed, the Cochrane Library, Wanfang Digital Journals, China National Knowledge Infrastructure (CNKI), and the VIP Database for Chinese Technical Periodicals. The search was completed on April 28, 2024. The reviewers independently performed literature screening, data extraction, and risk of bias (ROB) assessment using the revised Cochrane ROB tool. Data were analyzed using RevMan 5.4 and Stata 15.0 software.

**Results:**

Eight randomized controlled trials (RCTs) involving 550 patients (305 in the experimental group and 245 in the control group) were included. EA significantly reduced Pittsburgh Sleep Quality Index (PSQI) scores (SMD = −0.86, 95% CI [−1.24, −0.49], *p* < 0.001), Insomnia Severity Index (ISI) scores (SMD = −1.14, 95% CI [−1.59, −0.69], *p* < 0.001), sleep latency (SL) (SMD = −0.48, 95% CI [−0.73, −0.23], *p* < 0.001), and sleep disturbance (SDB) (SMD = −0.44, 95% CI [−0.73, −0.16], *p* = 0.002). EA also significantly lowered Hospital Anxiety and Depression Scale-Anxiety (HADS-Anxiety) scores (SMD = −0.59, 95% CI [−0.91, −0.26], *p* < 0.001) and Hospital Anxiety and Depression Scale-Depression (HADS-Depression) scores (SMD = −0.73, 95% CI [−1.06, −0.40], *p* < 0.001), while increasing total sleep time (TST) (SMD = 0.65, 95% CI [0.14, 1.17], *p* = 0.013). No significant differences were observed in the Athens Insomnia Scale (AIS), sleep duration (SD), sleep efficiency (SE), or sleep quality (SQ) scores between the EA and control groups.

**Conclusion:**

Electroacupuncture has shown promising potential in treating cancer-related insomnia by increasing total sleep time and reducing sleep disturbances. However, additional high-quality studies are necessary to validate these findings.

**Systematic review registration:**

https://www.crd.york.ac.uk/PROSPERO/display_record.php?RecordID=567567, Identifier CRD42024567567.

## 1 Introduction

Cancer-related insomnia is a common and significant complication affecting many cancer patients and survivors. Symptoms typically include difficulties falling asleep, frequent night awakenings, early morning awakenings, and excessive dreaming (1, 2). The prevalence of insomnia in cancer patients is estimated to exceed 60%, which is 2 to 3 times higher than that in the general population (3, 4). Over 40% of cancer patients report sleep disturbances following their diagnosis, and 20% continue to experience poor sleep quality up to 9 years after diagnosis (5, 6). Insomnia can severely impact quality of life and exacerbate pain, potentially increasing anxiety and depression in cancer patients (7). Furthermore, insomnia may adversely affect tumor progression by impairing immune responses (8, 9). Patients undergoing treatments such as surgery, radiotherapy, and chemotherapy are particularly vulnerable to sleep disorders due to the treatment’s side effects, which can further degrade their quality of life and prognosis. Thus, effectively addressing cancer-related insomnia is critical for enhancing patient well-being.

Medication is a common treatment for insomnia, but long-term use often leads to side effects such as withdrawal symptoms, cognitive impairment, and headaches, which can result in poor tolerance and drug dependence (10, 11). Additionally, sleeping pills may interact with chemotherapy drugs, potentially disrupting cancer treatment plans (12). Cognitive Behavioral Therapy (CBT), a non-pharmacological intervention, is an established effective treatment for insomnia (13, 14). However, CBT is time-consuming, complex, and requires a high degree of self-discipline, making adherence challenging for some patients (15).

Electroacupuncture (EA) is a therapeutic modality that combines traditional acupuncture techniques with electrical stimulation to modulate the nervous system. In EA, small electrical currents are applied to needles inserted at specific acupoints, stimulating the body’s neural pathways (16). Many studies have shown that electroacupuncture is beneficial in alleviating cancer symptoms, reducing side effects related to cancer treatment, and relieving cancer pain, fatigue, and insomnia, with minimal side effects (17–20).

Although several randomized controlled trials (RCTs) support EA’s efficacy in managing cancer-related insomnia, these studies are often limited by small sample sizes and lack robust evidence-based foundations. Additionally, many studies are small-scale and produce conflicting results. Thus, there is a clear need for an updated and comprehensive systematic review. This review aims to systematically analyze the current literature and conduct a meta-analysis to evaluate both the efficacy and safety of electroacupuncture in treating cancer-related insomnia.

## 2 Methods

### 2.1 Retrieval strategy

This review was conducted in accordance with the PRISMA (Preferred Reporting Items for Systematic Reviews and Meta-Analyses) guidelines (21). It has been registered with PROSPERO (ID: CRD42024567567). The search strategy covered seven databases from their inception up to April 28, 2024, with the aim of identifying relevant articles regardless of language. The databases searched included EMBASE, Web of Science, PUBMED, the Cochrane Library, Wanfang Digital Journals, China National Knowledge Infrastructure (CNKI), and the VIP Database for Chinese Technical Periodicals. The key search terms were “Electroacupuncture” and (insomnia or “Sleep disorder” or “Sleep Initiation and Maintenance Disorders” or “Sleep Initiation Dysfunction”) and (Cancer or Neoplasms or Tumor or Malignancy). Search strategies were customized for each database. Additionally, we manually reviewed the reference lists of the included studies to identify any relevant documents that might not have been captured through the database searches.

### 2.2 Inclusion and exclusion criteria

The inclusion criteria for this study were randomized controlled trials (RCTs) involving individuals with cancer and sleep disorders. The intervention in these trials must have included an experimental group receiving electroacupuncture (EA) compared to a control group receiving either no intervention or alternative interventions. The primary outcome measures assessed included Pittsburgh Sleep Quality Index (PSQI) scores, total sleep time (TST), Athens Insomnia Scale (AIS) scores, and Insomnia Severity Index (ISI) scores, both before and after the intervention. Exclusion criteria included non-randomized controlled trials, animal studies, conference abstracts, letters to the editor, case reports, and studies with insufficient data or irrelevant outcomes. Additionally, patients with a preoperative PSQI score greater than 7 or an AIS score greater than 6 were excluded due to the presence of non-cancer-related sleep disorders.

### 2.3 Data extraction

In accordance with the pre-defined inclusion and exclusion criteria, two independent researchers performed a comprehensive screening of the literature, followed by data extraction and entry. Their findings were then cross-verified. In the event of discrepancies, a consensus was reached through discussions that involved a third researcher. The extracted data covered several key aspects: the first author’s name, publication year, country of origin, participant characteristics (including sample size, age distribution, and gender composition), study design details (such as cancer type and stage), electroacupuncture protocol (including treatment duration and frequency), and outcome measurements. To facilitate data extraction, a standardized information extraction form was used. This form included fields for the first author’s name, country of research, publication year, participant characteristics, study design details (sample size, age, gender, cancer type, and stage), electroacupuncture plan (treatment course, duration, and frequency), and outcome measurements, among other relevant information.

### 2.4 ROB evaluation

The quality of the studies included in the analysis was independently assessed by two review authors. In cases of disagreement, a third reviewer was consulted to reach a resolution. The researchers employed the bias assessment tool described in the Cochrane Handbook for Systematic Reviews of Interventions (version 5.1.0.19) to evaluate the quality of the studies.

This tool evaluates studies based on seven criteria: random sequence generation, allocation concealment, blinding of participants and personnel, blinding of outcome assessors, completeness of data and results, selective reporting of study results, and other sources of bias. Each criterion involves multiple aspects to be assessed for each study, leading to a thorough evaluation of study quality. A risk of bias map is then generated to visually represent the assessment outcomes.

### 2.5 Grade of evidence

To determine the quality of our results, we selected the Graded Recommendations Assessment Development and Evaluation (GRADE) system to evaluate the evidence for methodological quality (22). We considered five factors that could reduce the quality of the evidence, including study limitations, inconsistent findings, inconclusive direct evidence, inaccurate or wide confidence intervals, and publication bias. In addition, three factors that could reduce the quality of evidence were reviewed, namely effect size, possible confounding factors, and dose-effect relationships. A comprehensive description of the quality of evidence for each parameter data is provided, (for detailed information, please refer to Supplementary Table S1).

### 2.6 Statistical analysis

Meta-analysis was performed using statistical software Stata 15.0 and RevMan 5.4. Continuous variables were summarized using the standardized mean difference (SMD) with corresponding 95% confidence intervals (CI). The degree of statistical heterogeneity among the studies was assessed using the I^2^ statistic, in accordance with the Cochrane Handbook.

When the heterogeneity among the studies was not substantial (I^2^ ≤ 50%), a fixed-effects model was applied. Conversely, when significant heterogeneity was present (I^2^ > 50%), a random-effects model was used. Sensitivity analysis was conducted to evaluate the robustness of the meta-analysis results. Due to the small number of included studies (*n* < 10), the Egger test was employed to assess publication bias.

## 3 Results

### 3.1 Literature screening

**Figure 1:**
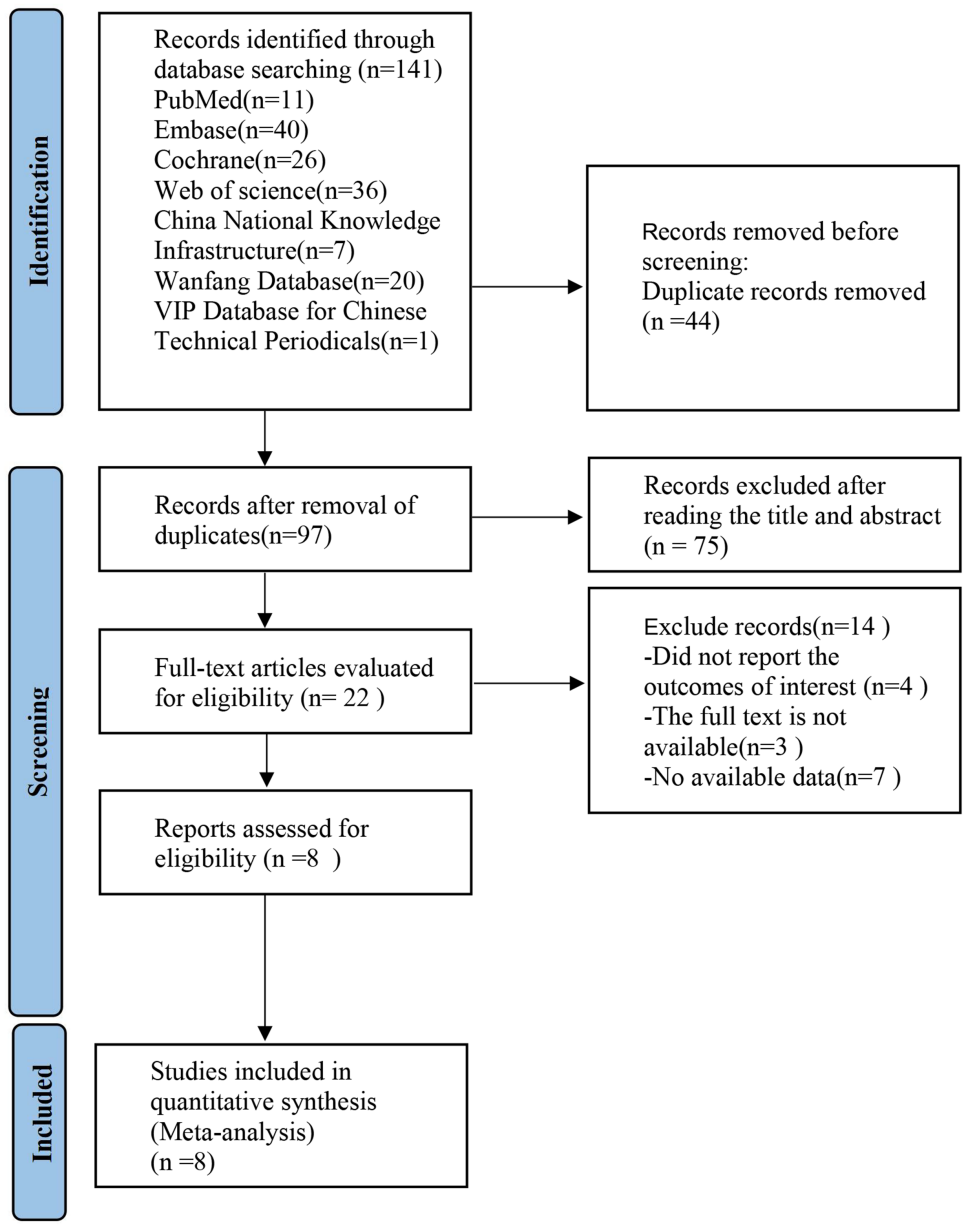
PRISMA flow diagram showing the screening and selection process of reports to be included in the meta-analysis. PRISMA, preferred reporting items for systematic review and meta-analysis.

A literature search identified 141 articles, of which 44 were duplicates. After screening titles and abstracts, 75 articles were excluded for being irrelevant. The remaining 22 clinical trials were reviewed in full for further evaluation. Of these, 14 were excluded for various reasons. Ultimately, 8 studies were included in this review, as shown in Figure 1.

### 3.2 The basic characteristics table of the included literature

A total of 8 studies were included in the review (23–30), with sample sizes ranging from 16 to 158 participants. In total, 550 patients were involved: 305 in the experimental group and 245 in the control group. The studies were published between 2014 and 2023.

Of the included studies, six were published in English and two in Chinese. The studies originated from various countries: four from China, two randomized controlled trials from the United States, one from Canada, and one from Korea. The patients in these studies had a range of cancer types and treatment scenarios. The key characteristics of the included studies are summarized in Table 1.

**Table 1:** Characteristics of randomized controlled trials included in the meta-analysis.

Study	Year	Country	Sample size		Gender (M/F)	Cancer Stage	Insomnia score (Baseline mean)		Mean age (years)		Intervention		Outcome
			EG	CG			EG	CG	EG	CG	EG	CG	
Lee	2022	Korea	8	8	5/11	Breast, Thyroid, Gastrointestinal I-IV	PSQI: 14.25	PSQI: 13.00	57.63	61.38	EA: 4HZ, continuous wave,30 min, 2–3 times a week x 4 weeks.	UC: cognitive behavior therapy, or hypnotics, or no treatment	ISI, PSQI, SL, TST, SE,AE
Lufei	2016	China	50	50	59/41	Lung cancer IIIb-IV	PSQI: 15.26	PSQI: 14.95	54.93	58.09	EA: 10HZ, dense wave, 30 min, once daily x 4 weeks.	UC: 10 mg of zolpidem orally each night prior to going to bed	PSQI,SAS, SDS
Yinghao	2022	China	30	30	39/21	Gastric cancer	PSQI: 3.12	PSQI: 3.34	66.00	68.00	EA: 2/100 Hz, disperse-dense wave, 30 min, once in total before surgery.	UC: no treatment	PSQI,AIS
Garland	2016	Canada	30	28	0/58	breast cancer 0-III	PSQI: 9.10	PSQI: 8.40	52.90	50.40	EA: 2 Hz 30 min, a total of 10 treatments over 8 weeks.	Gabapentin: total daily dose of 900 mg	PSQI,SD,SDB, SL,SE,SQ
Yang	2023	America	110	48	42/116	Breast cancer, Prostate cancer, Colorectal cancer, Lymphoma cancer, Melanoma cancer, Lung cancer,	PSQI: 10.40	PSQI: 10.10	60.20	61.00	EA: 2 Hz, 30 min, 10 times a week x 10 weeks.	UC: received standard care for their pain	PSQI,SE,SQ, SL,SD,SDB
Wang	2023	China	40	43		Gastrointestinal Tumor	PSQI: 1.12	PSQI: 1.16	58.20	60.44	TEAS: 2/10 Hz, dense-disperse wave, 30 min, 3 times for total.	sham TEAS	PSQI,AIS
Mao	2014	America	22	23	0/45	Breast Cancer I-III	PSQI: 8.70	PSQI: 8.30	57.50	60.60	EA: 2 Hz 30 min, a total of 10 treatments over 8 weeks.	WLC	PSQI, HADS-Anxiety HADS-Depression
Zhang	2021	China	15	15	0/30	Breast Cancer I-IV	PSQI: 12.50	PSQI: 11.30	52.50	52.70	EA: 2-5 Hz, continuous wave 25 min, 2 times a week x 6 weeks.	WLC	ISI,SL, PSQI, TST SE, HADS-Anxiety, HADS-Depression, AE

AE, adverse effect; EG, Electroacupuncture group; CG, Usual-care group; M/F, Male/Female; UC, Usual-care; ISI, insomnia severity index; PSQI, Pittsburgh sleep quality index; TST, total sleep time; SE, sleep efficiency; SQ, sleep quality; SL, sleep latency; SD, sleep duration; SDB, sleep disturbances; AIS, Athens insomnia scale; WASO, wake time after sleep onset; SAS, self-rating anxiety scale; SDS, self-rating depression scale; HADS, Hospital Anxiety and Depression Scale; TEAS, transcutaneous electrical acupoint stimulation; WLC, wait-list control.

### 3.3 ROB assessment

**Figure 2:**
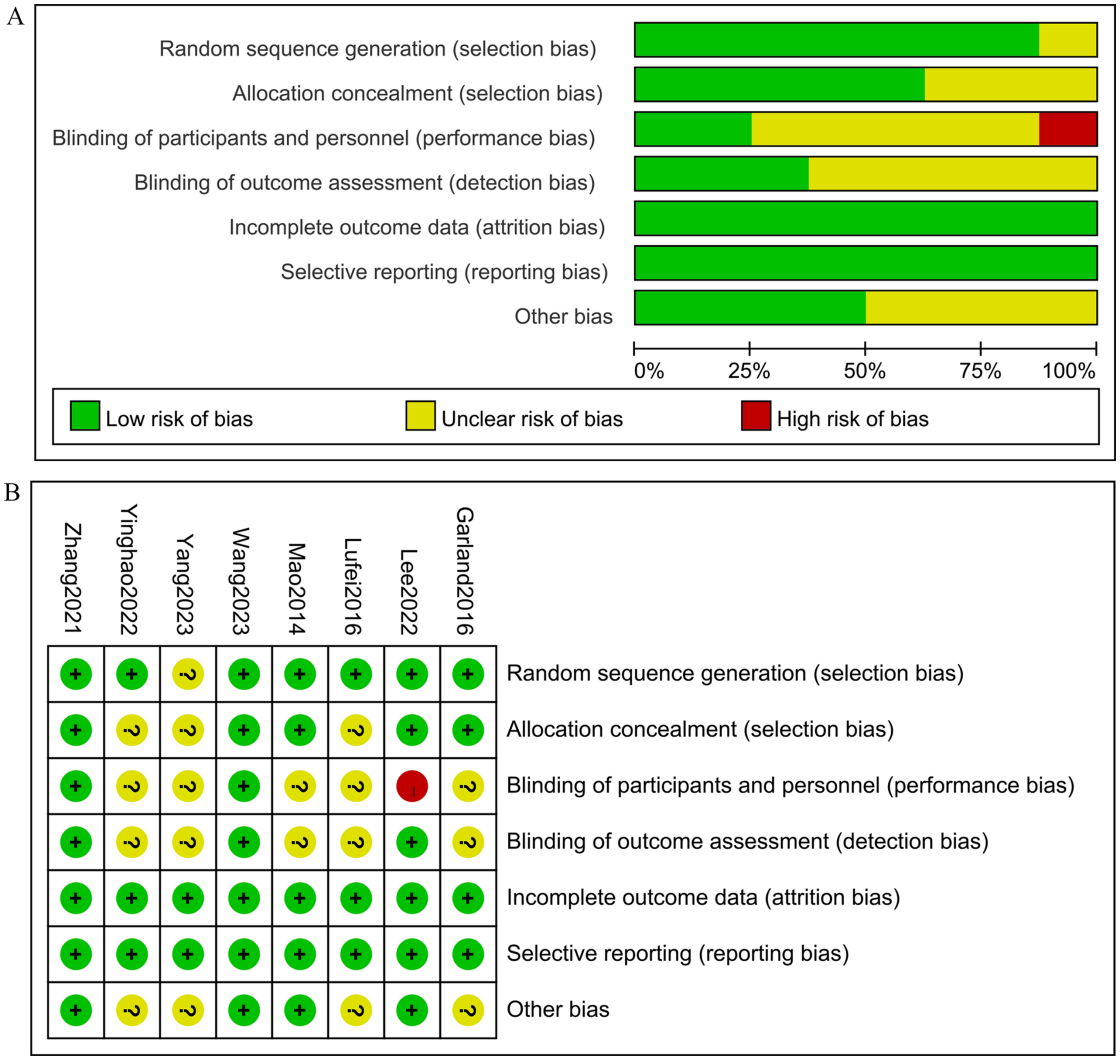
The figure represents the risk of bias assessment for the studies selected in the meta-analysis. **(A)** Risk of bias graph, **(B)** Risk of bias summary.

All 8 randomized controlled trials (RCTs) (23–30) employed randomization methods. However, one study (27) did not specify the randomization technique used. Five studies (23, 26, 28–30) utilized sealed envelope methods for allocation concealment, while the remaining studies did not provide information on allocation concealment. Two studies (28, 30) described blinding procedures for both participants and assessors. Study (23) only implemented blinding for outcome evaluators and received high risk of bias (ROB) ratings for blinding of implements and participants. All eight studies reported on withdrawals and dropouts. Five studies had at least one area rated as uncertain in the ROB assessment due to insufficient information. The risk of bias for the studies is illustrated in Figure 2.

### 3.4 Meta-analysis

#### 3.4.1 Meta-analysis of change in PSQI score

**Figure 3:**
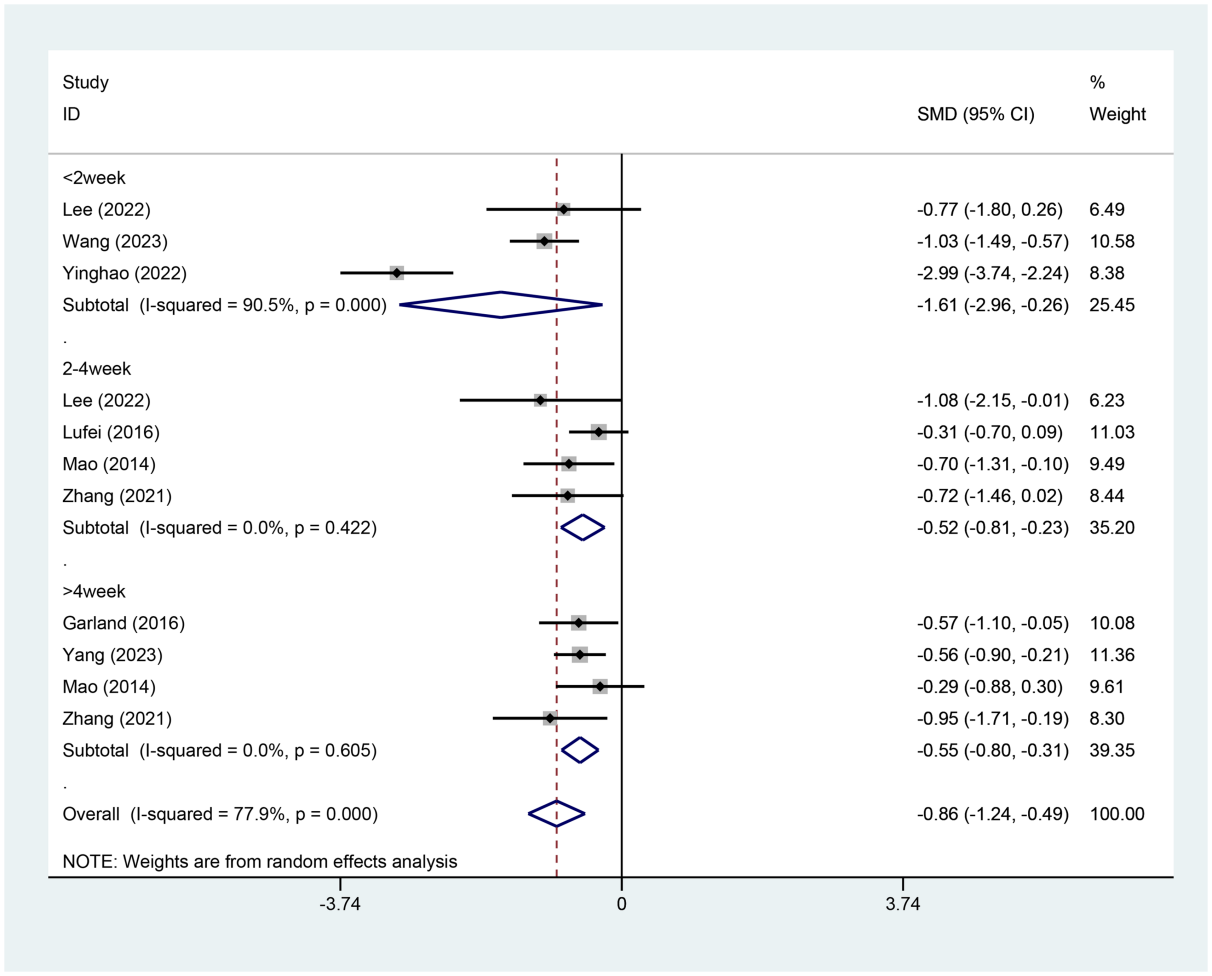
Forest plot for PSQI score. PSQI, Pittsburgh sleep quality index; CI, confidence interval; SMD, standardized mean difference.

All eight studies reported the aggregate Pittsburgh Sleep Quality Index (PSQI) scores. The experimental group included 350 patients, while the control group comprised 291 individuals. A heterogeneity test revealed an I^2^ value of 77.90% with a *p*-value of 0.00, indicating substantial heterogeneity. Consequently, a random-effects model was applied for data analysis. The results of the analysis showed a standardized mean difference (SMD) of −0.86 (95% CI [−1.24, −0.49], *p* = 0.00), suggesting that electroacupuncture (EA) significantly reduced PSQI scores compared to the control group, as depicted in Figure 3. Sensitivity analysis using a stepwise exclusion method did not alter the results, indicating a low level of sensitivity and demonstrating that the findings are robust. These results are presented in Supplementary Figure S1. The Egger test (*p* = 0.187) indicated no significant publication bias, as shown in Supplementary Figure S2.

#### 3.4.2 Meta-analysis of change in AIS score

**Figure 4:**
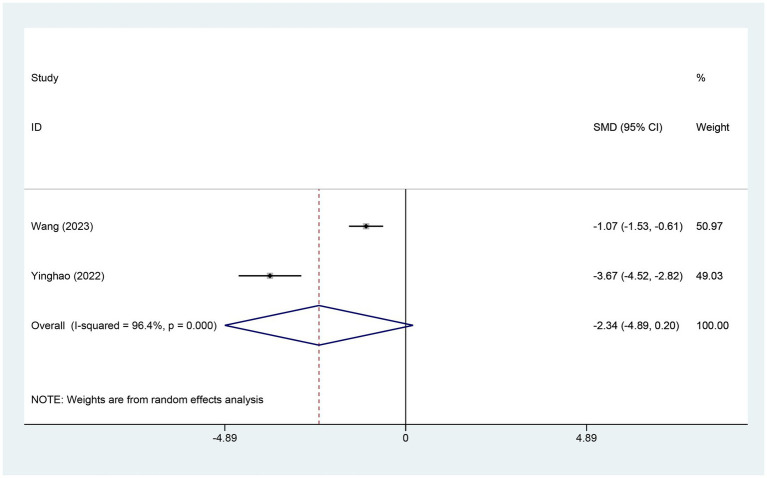
Forest plot for AIS score. AIS, Athens insomnia scale; CI, confidence interval; SMD, standardized mean difference.

Two studies reported variations in the AIS score. The experimental group comprised 70 patients, while the control group included 73 patients. A heterogeneity test revealed an I^2^ of 96.40% with a *p*-value of 0.00, indicating high heterogeneity. Consequently, a random-effects model was used for data analysis. The analysis results (SMD = −2.34,95% CI [−4.89, 0.20],*p* = 0.071) indicated that the effect of electroacupuncture (EA) on reducing AIS scores was similar between the experimental and control groups, as shown in Figure 4. Sensitivity analysis, conducted using the method of exclusion, did not alter the results, indicating that the findings are stable and that the analysis is robust. These results are detailed in Supplementary Figure S3.

#### 3.4.3 Meta-analysis of change in ISI score

**Figure 5:**
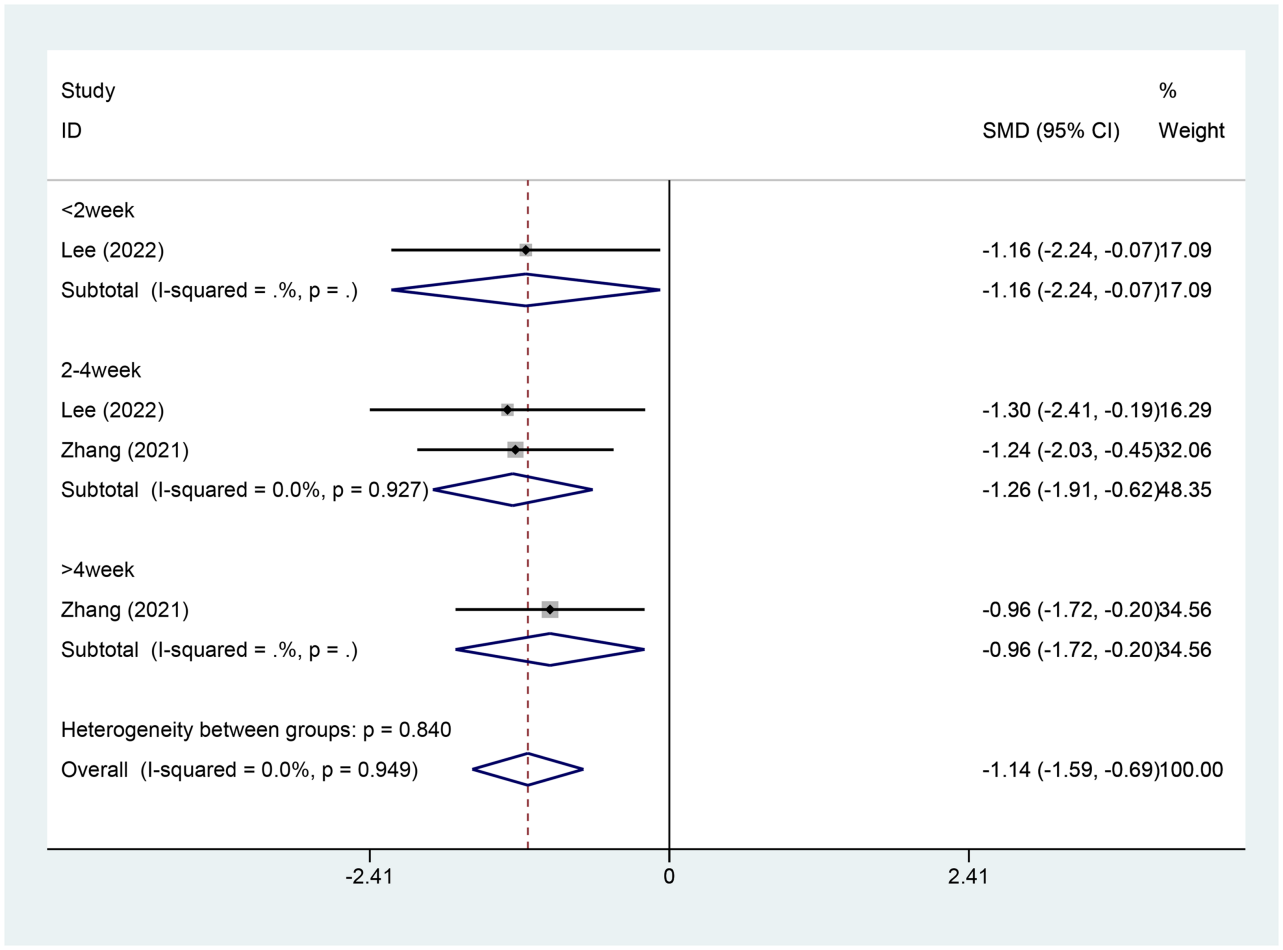
Forest plot for ISI score. ISI, insomnia severity index; CI, confidence interval; SMD, standardized mean difference.

Two studies reported changes in the ISI score, with 46 patients in the electroacupuncture (EA) group and 46 in the control group. A heterogeneity test revealed an I^2^ value of 0.00% and a *p*-value of 0.949, indicating no significant heterogeneity. Consequently, a fixed-effects model was used for data analysis. The analysis results (SMD = −1.14,95% CI [−1.59, -0.69],*p* = 0.00) implied that EA led to a significant reduction in ISI scores compared to the control group, as depicted in Figure 5. The Egger test (*p* = 0.462) was conducted to evaluate publication bias, and the results indicated no evidence of publication bias, as shown in Supplementary Figure S4.

#### 3.4.4 Meta-analysis of change in SL score

**Figure 6:**
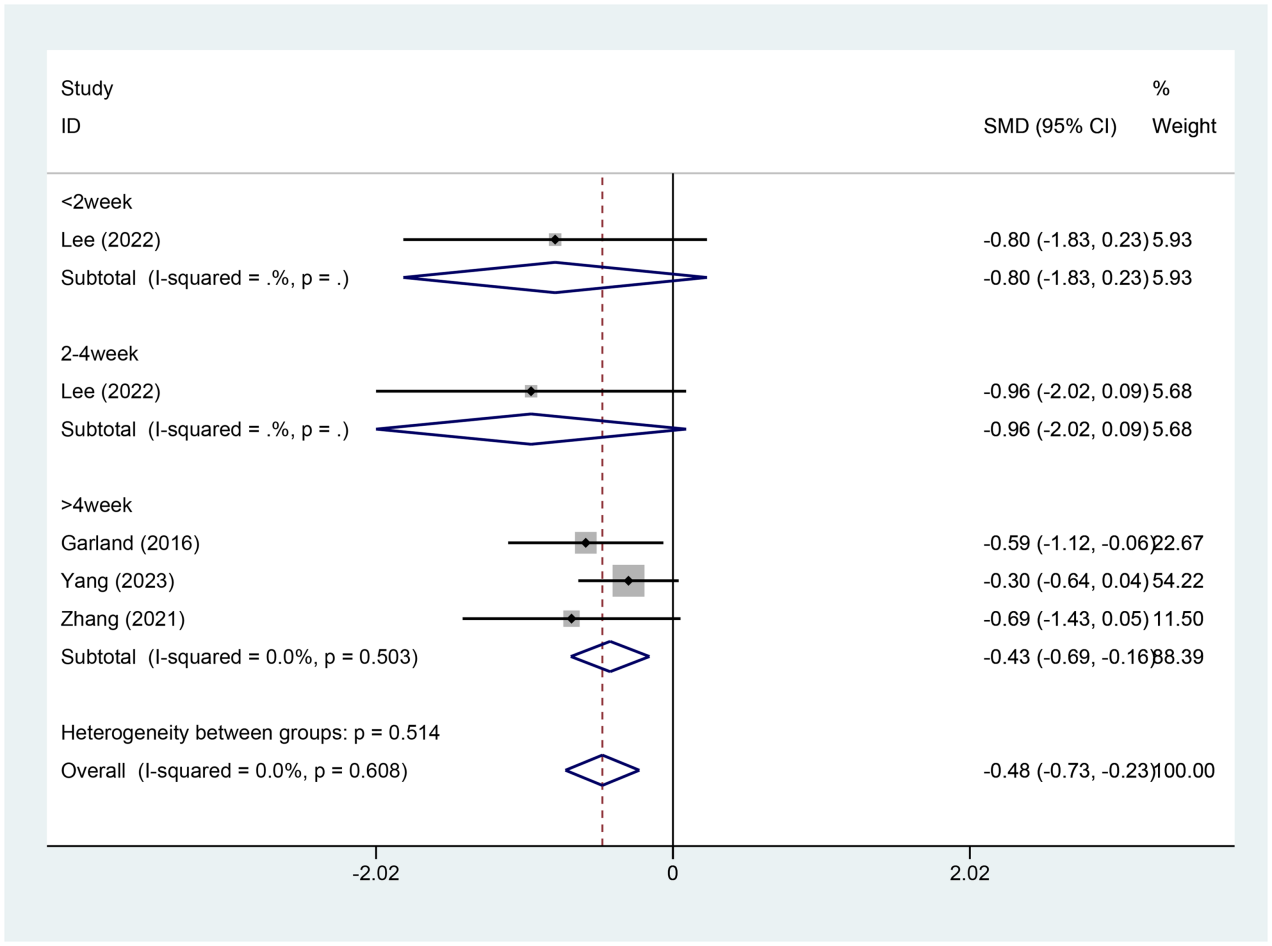
Forest plot for SL score. SL, sleep latency; CI, confidence interval; SMD, standardized mean difference.

Four studies reported on the alterations in sleep latency (SL) scores. There were 171 patients in the EA group and 107 in the control group. A heterogeneity test was conducted with an I2 value of 0.00% and a *p* value of 0.608. As a result, a fixed effects model was employed for data analysis. The analysis results (SMD = −0.48,95% CI [−0.73, −0.23], *p* = 0.00) implied that EA could lead to a significant reduction in the SL scores in contrast to the control group, as depicted in Figure 6. The Egger test was adopted (*p* = 0.011 < 0.05), and the results suggested the possible existence of publication bias, as shown in Supplementary Figure S5.

#### 3.4.5 Meta-analysis of change in SE score

**Figure 7:**
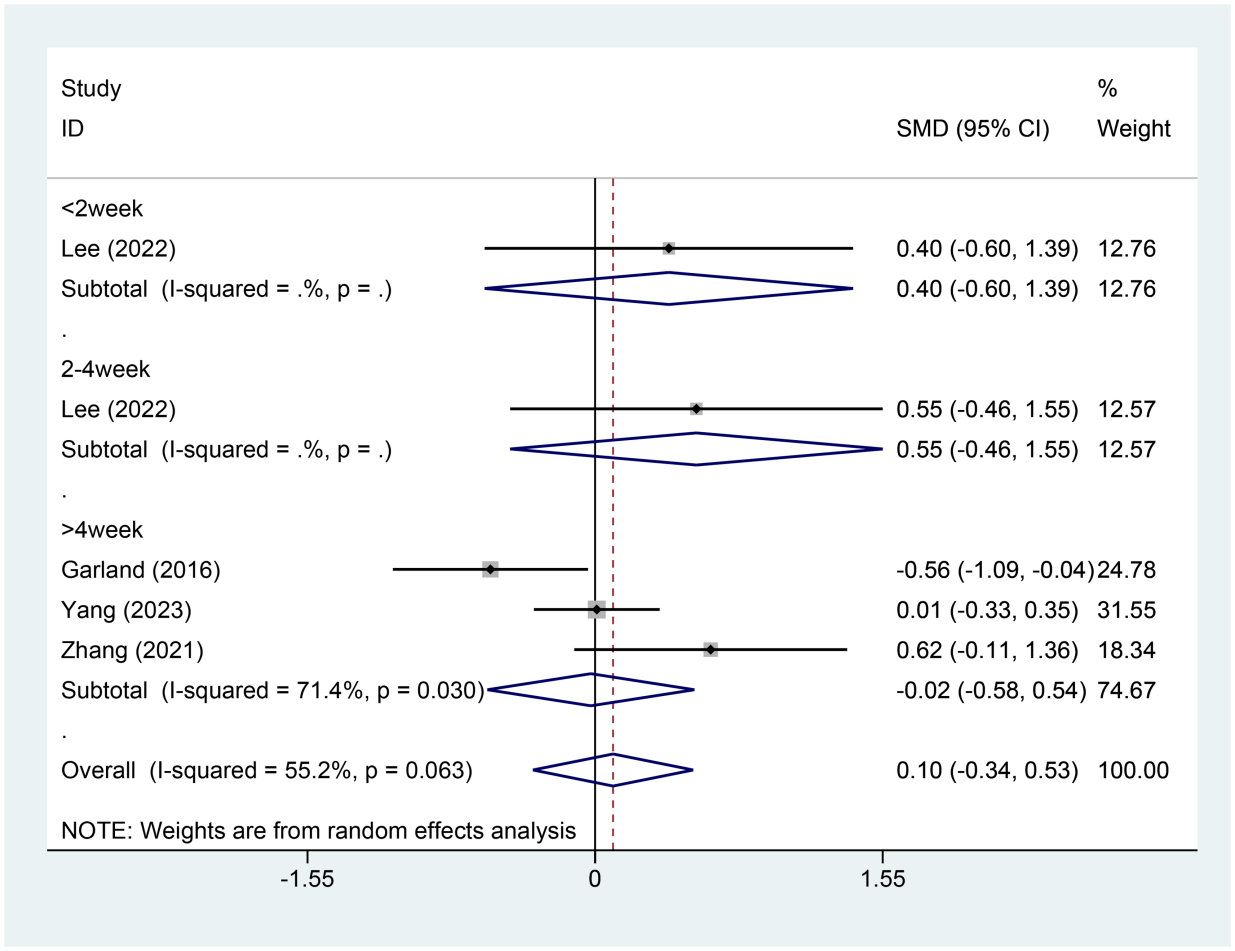
Forest plot for SE score. SE, sleep efficiency; CI, confidence interval; SMD, standardized mean difference.

Four studies referred to the alterations in sleep efficiency (SE) scores. There were 171 patients within the EA group and 107 within the control group. A heterogeneity test was carried out, resulting in an I2 value of 55.2% and a *p* value of 0.063. Consequently, a random effects model was employed for data analysis. The analysis results (SMD = 0.10,95% CI [−0.34, −0.53], *p* = 0.661) suggested that the effect of EA on changing SE was similar between the experimental and control groups, as shown in Figure 7. Sensitivity analysis was performed using a one-by-one exclusion method. The results remained consistent, with minimal sensitivity, indicating that the findings are stable. For details, see Supplementary Figure S6. Egger test was adopted (*p* = 0.405 > 0.05), and the results indicated that no publication bias was found, as shown in Supplementary Figure S7.

#### 3.4.6 Meta-analysis of change in TST score

**Figure 8:**
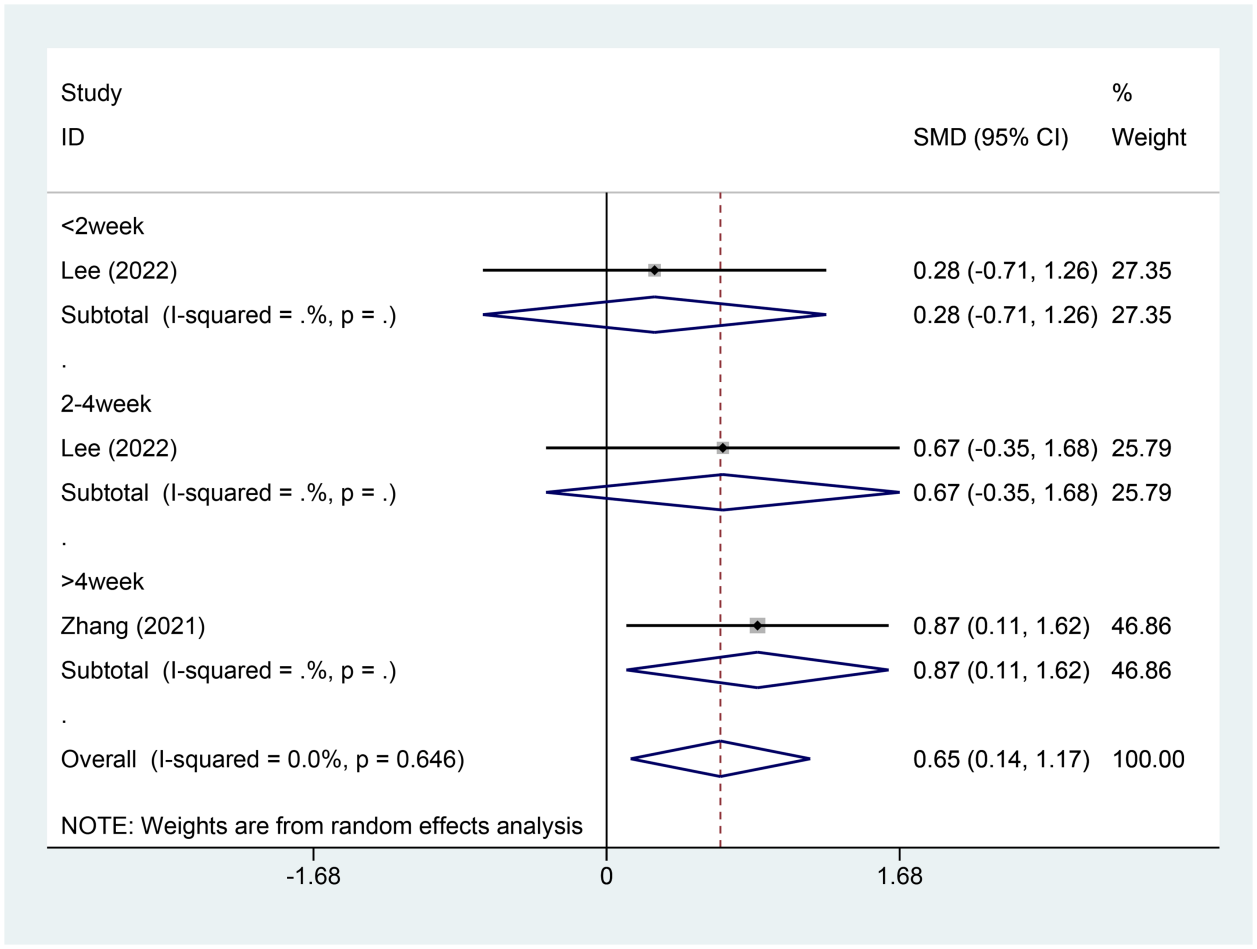
Forest plot for TST score. TST, total sleep time; CI, confidence interval; SMD, standardized mean difference.

Two studies brought up the alterations in total sleep time (TST) scores. There were 31 patients in the EA group and an equal number in the control group. A heterogeneity test was conducted, yielding an I2 value of 0.0% and a *p* value of 0.648. As a consequence, a fixed effects model was utilized for data analysis. The analysis outcomes (SMD = 0.65,95% CI [0.14, 1.17], *p* = 0.013) implied that EA could lead to an increase in the TST scores in comparison to the control group, as illustrated in Figure 8. Egger test was adopted (*p* = 0.45 > 0.05), and the result indicated that no publication bias was found, as shown in Supplementary Figure S8.

#### 3.4.7 Meta-analysis of change in SD score

**Figure 9:**
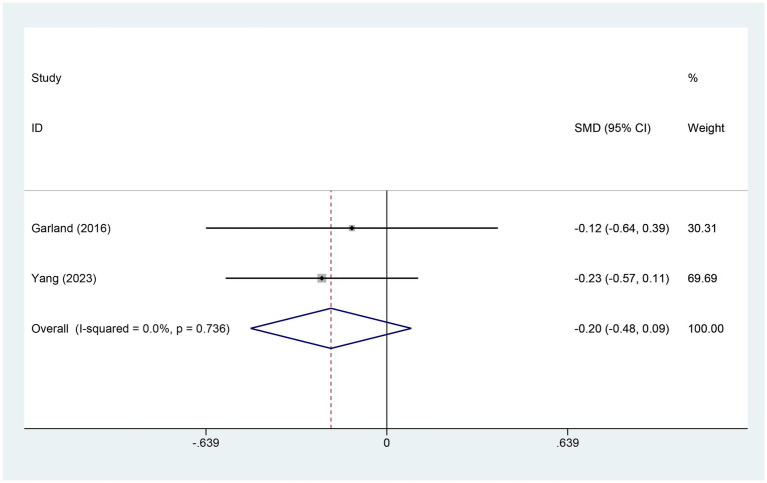
Forest plot for SD score. SD, sleep duration; CI, confidence interval; SMD, standardized mean difference.

Two studies referred to the alterations in sleep disturbance (SD) scores. There were 140 patients within the EA group and 76 within the control group. A heterogeneity test was carried out, resulting in an I2 value of 0.0% and a p value of 0.736. Consequently, a fixed effects model was employed for data analysis. The analysis results (SMD = −0.20, 95% CI [−0.48, 0.09], *p* = 0.173) indicated that the effect of EA on changing SD was similar between the experimental and control groups, as shown in Figure 9.

#### 3.4.8 Meta-analysis of change in SDB score

**Figure 10:**
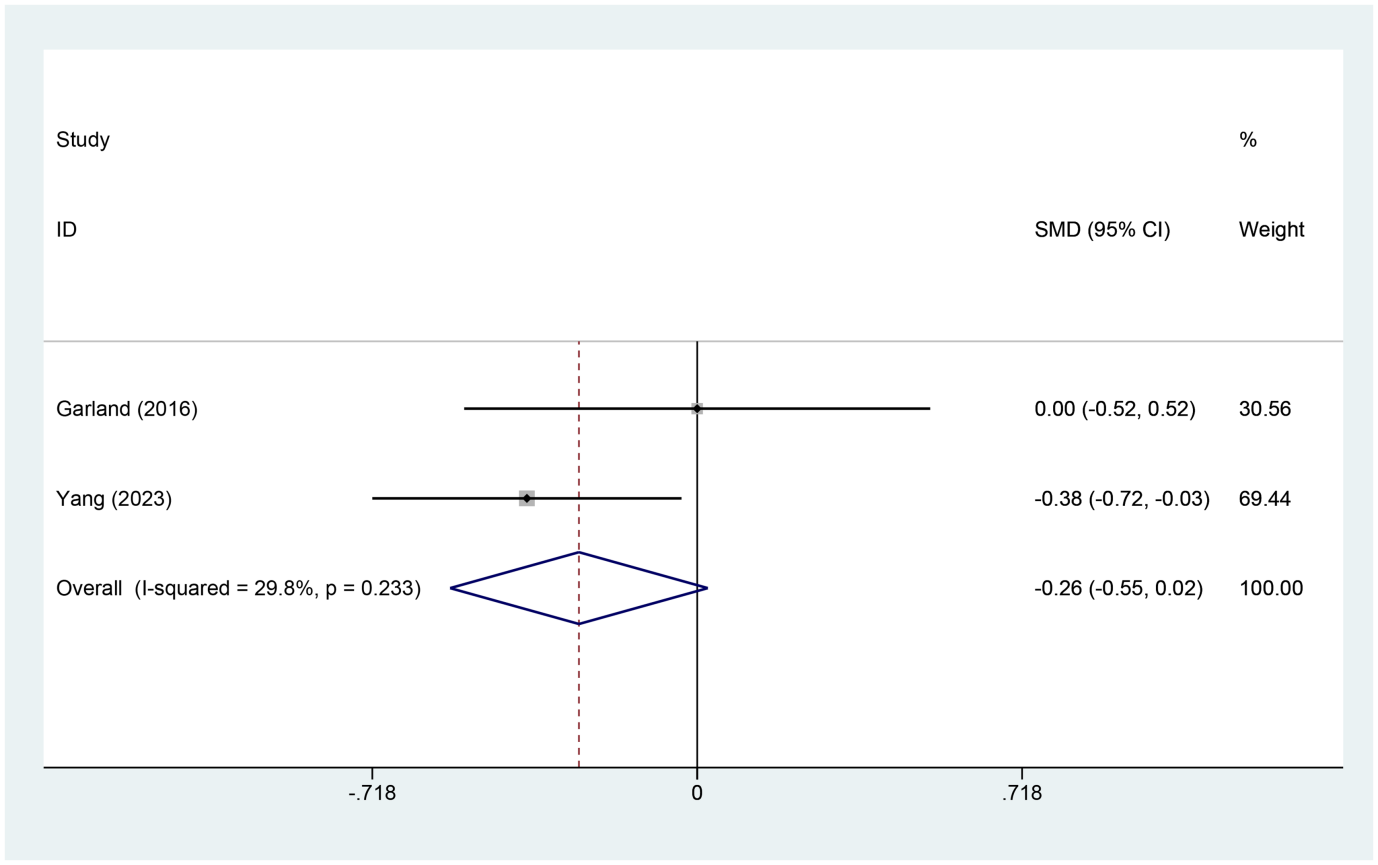
Forest plot for SDB score. SDB, sleep disturbances; CI, confidence interval; SMD, standardized mean difference.

Two studies assessed variations in SDB scores. The electroacupuncture (EA) group included 140 patients, while the control group had 76 patients. A heterogeneity test showed an I^2^ value of 29.3% with a *p*-value of 0.234, indicating low heterogeneity. Consequently, a fixed-effects model was used for data analysis. The analysis results (SMD = −0.44, 95% CI [−0.73, −0.16], *p* = 0.002) suggest that electroacupuncture (EA) significantly reduced SDB scores compared to the control group, as illustrated in Figure 10.

#### 3.4.9 Meta-analysis of change in SQ score

**Figure 11:**
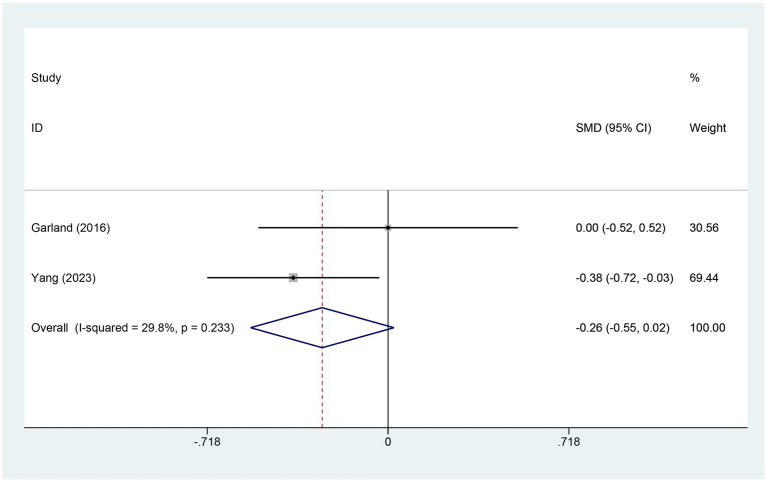
Forest plot for SQ score. SQ, sleep quality; CI, confidence interval; SMD, standardized mean difference.

Two studies examined changes in sleep quality (SQ) scores. The electroacupuncture (EA) group comprised 140 patients, while the control group included 76 patients. A heterogeneity test yielded an I^2^ value of 29.8% and a p-value of 0.233, indicating low heterogeneity. Consequently, a fixed-effects model was used for data analysis. The analysis results (SMD = −0.26, 95% CI [−0.55, 0.02], *p* = 0.072) suggest that the effect of electroacupuncture (EA) on sleep quality is similar to that of the control group, as shown in Figure 11.

#### 3.4.10 Meta-analysis of change in HADS-anxiety score

**Figure 12:**
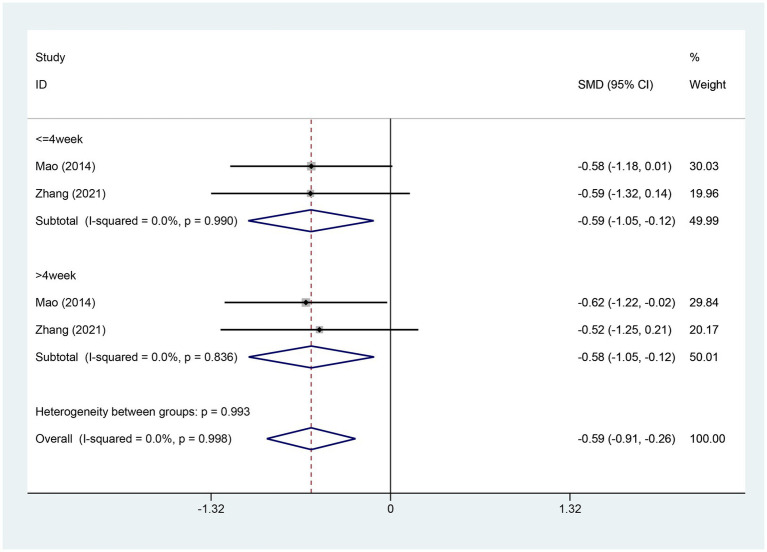
Forest plot for HADS-Anxiety score. HADS-Anxiety, Hospital Anxiety and Depression Scale anxiety; CI, confidence interval; SMD, standardized mean difference.

Two studies reported on the changes in the score of HADS-Anxiety. The experimental group comprised 74 cases while the control group contained 76 cases. A heterogeneity test was carried out, with an I2 value of 0.0% and a *p* value of 0.998. Consequently, a fixed effects model was utilized for data analysis. The analysis results (SMD = −0.59, 95% CI [−0.91, −0.26], *p* = 0.000) indicate that EA significantly reduces HADS-Anxiety scores compared to the control group, as shown in Figure 12. Egger test was adopted (*p* = 0.353 > 0.05), and the result indicated that no publication bias was found, as shown in Supplementary Figure S9.

#### 3.4.11 Meta-analysis of change in HADS-depression score

**Figure 13:**
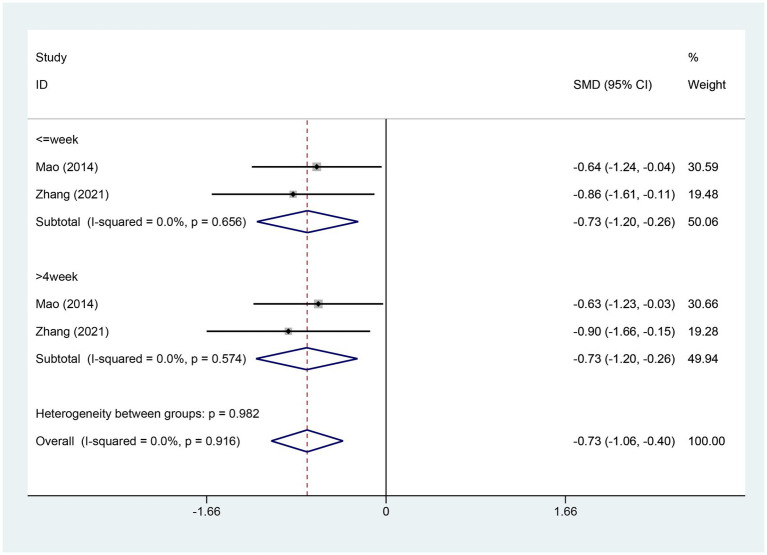
Forest plot for HADS-Depression score. HADS-Depression, Hospital Anxiety and Depression Scale Depression; CI, confidence interval; SMD, standardized mean difference.

Two studies provided details about the alterations in the HADS-Depression score. There were 74 cases within the experimental group and 76 cases within the control group. A heterogeneity test was carried out, resulting in an I2 value of 0.0% and a p value of 0.916. Consequently, a fixed effects model was employed for data analysis. The analysis results (SMD = −0.73, 95% CI [−1.06, −0.40], p = 0.000) indicate that EA significantly reduces HADS-Depression scores compared to the control group, as depicted in Figure 13.The Egger test was adopted (*p* = 0.006 < 0.05), and the results suggested the possible existence of publication bias, as shown in Supplementary Figure S10.

## 4 Discussion

This study conducted a systematic review and meta-analysis to evaluate the efficacy and safety of electroacupuncture for treating cancer-related insomnia. A total of eight trials involving 550 participants were included. Electroacupuncture was used as the intervention in the treatment groups across all studies, while control groups received sham acupuncture, no treatment, or usual care. To the best of our knowledge, this is the first meta-analysis examining the application of electroacupuncture for cancer-related insomnia. The combined results indicate that electroacupuncture significantly improved sleep quality in patients with cancer-related insomnia, with no serious adverse events reported.

The pathogenesis of cancer-related insomnia remains unclear. However, modern medicine suggests that sleep disorders in cancer patients may be linked to disruptions in biological rhythms, such as immune response and neuroregulation (31, 32). Additionally, cancer-related surgical treatments can induce local and systemic inflammation (33), leading to the prolonged activation of inflammatory cytokines and chemokines, which may adversely affect the body (34). Inflammatory cytokines can interact with the central nervous system (CNS) through neural and cellular pathways, resulting in alterations in CNS function and disruption of sleep regulation (35–37). This disruption is attributed to various factors that impair the function of the hypothalamic–pituitary–adrenal (HPA) axis and the circadian rhythm (38, 39). As our understanding of the pathophysiology of sleep disorders improves, non-pharmacological therapies for cancer-related sleep disorders are expected to play an increasingly crucial role in improving sleep quality and alleviating symptoms.

Acupuncture, which may help prevent the exacerbation of insomnia during cancer treatment, and its potential effectiveness in addressing insomnia is probably associated with its impacts on the hypothalamic–pituitary–adrenal (HPA) axis, a crucial neuroendocrine system implicated in sleep modulation and frequently observed to be overactive in people with insomnia, as well as neurotransmitters. (40–42). Research indicates that acupuncture holds significant potential for addressing cancer-related insomnia in cancer patients and survivors (43–47). EA has shown promise in increasing levels of *γ*-aminobutyric acid, dopamine, and melatonin, which are essential for improving sleep quality (16, 19, 48). EA also impacts the autonomic nervous system by reducing nervous system overexcitation and regulating biological rhythms, which supports better sleep (49–51). Furthermore, during cancer treatment, patients often experience pain, depression, insomnia, hot flashes, or excessive sweating, and acupuncture has been shown to effectively alleviate these symptoms (20, 52, 53).

Previous reviews (17, 46, 54, 55) have identified acupuncture as a beneficial intervention for alleviating cancer-related insomnia. However, the evidence supporting the use of electroacupuncture (EA) for this condition remains inconclusive. This uncertainty arises from limitations such as a limited number of clinical trials, small sample sizes, and varying methodological quality. Unlike previous reviews, this study conducted a more comprehensive search, incorporating recently published trials. The aim was to analyze subjective sleep measures and evaluate the efficacy of EA in treating cancer-related insomnia.

The analysis demonstrates that EA significantly reduces Pittsburgh Sleep Quality Index (PSQI) and Insomnia Severity Index (ISI) scores, indicating an improvement in cancer-related insomnia. Furthermore, treatment durations of 0–2 weeks, 2–4 weeks, and more than 4 weeks all yielded favorable outcomes. The effectiveness of EA may be attributed to its ability to enhance local stimulation, promote regular blood circulation, and regulate neural activity rhythms, potentially extending the treatment effects. Since this study did not investigate longer treatment durations, the optimal length of EA treatment remains to be determined. The review also found that EA can reduce sleep latency (SL) and sleep-disordered breathing (SDB) scores while increasing total sleep time (TST) scores. However, there was no statistical significance observed for scores on the Athens Insomnia Scale (AIS), sleep disturbance (SD), sleep efficiency (SE), and sleep quality (SQ). This lack of significance may be due to the limited data available on these measures in the included studies. Notably, EA showed a clear effect on reducing Hospital Anxiety and Depression Scale (HADS) anxiety and depression scores. However, due to the limited number of studies included in the meta-analysis, this finding requires further investigation.

Regarding adverse events, both the EA and control groups reported similar types, such as bleeding, hematoma, and acupoint pain. These incidents were generally mild to moderate, with no significant differences between the groups. EA offers advantages over manual acupuncture by performing repetitive mechanical actions and allowing for more precise control of stimulation levels, thereby enhancing the efficiency and standardization of acupuncture practices. Thus, electroacupuncture may be considered a viable therapeutic option for cancer patients experiencing insomnia.

Despite promising results, several limitations must be considered. First, significant heterogeneity was observed across studies, likely due to clinical differences such as variations in cancer types, treatment regimens, and control interventions. Additionally, small sample sizes in some subgroups may have reduced statistical power and limited generalizability. The methodological quality of the included studies also varied, potentially introducing bias, although sensitivity analyses indicated stable results despite some heterogeneity. Furthermore, the studies primarily used subjective measures, such as the Pittsburgh Sleep Quality Index (PSQI), which may not fully capture the complexity of cancer-related insomnia. Future studies should incorporate objective sleep assessments, such as actigraphy or polysomnography, to provide a more comprehensive evaluation of electroacupuncture’s effects on sleep quality.

## 5 Conclusion

Electroacupuncture appears to be effective in improving cancer-related insomnia, as evidenced by significant reductions in the PSQI, ISI, and AIS scores. However, the quality of the evidence varied across different outcomes. While electroacupuncture shows promise in addressing insomnia associated with cancer, the overall evidence is still limited and subject to variability. Future research should focus on studies with rigorous methodological designs, larger sample sizes, and multi-center involvement to strengthen the generalizability and reliability of the findings. Moreover, further exploration of the underlying mechanisms through which electroacupuncture alleviates cancer-related insomnia is crucial for providing more robust and objective clinical evidence. This will help inform clinical practice and guide the development of standardized treatment protocols.

## Data Availability

The original contributions presented in the study are included in the article/Supplementary material, further inquiries can be directed to the corresponding authors.
