# Supplementary material for: Efficacy of electroacupuncture for insomnia in cancer patients: a systematic review and meta-analysis
Source: Front Neurol. 2025 Feb 10;16:1512052. doi: 10.3389/fneur.2025.1512052 (PMC11847688; doi:10.3389/fneur.2025.1512052)
Supplement: Supplementary file 1 [file Supplementary_file_1.docx]

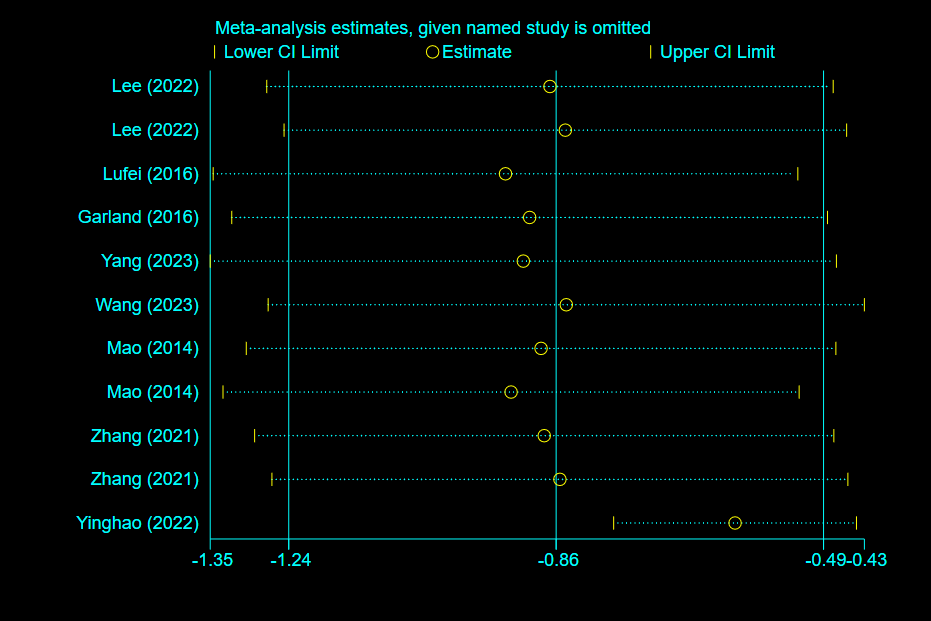


Figure 1 Sensitivity analysis for PSQI score. PSQI: Pittsburgh sleep quality index; CI: confidence interval.


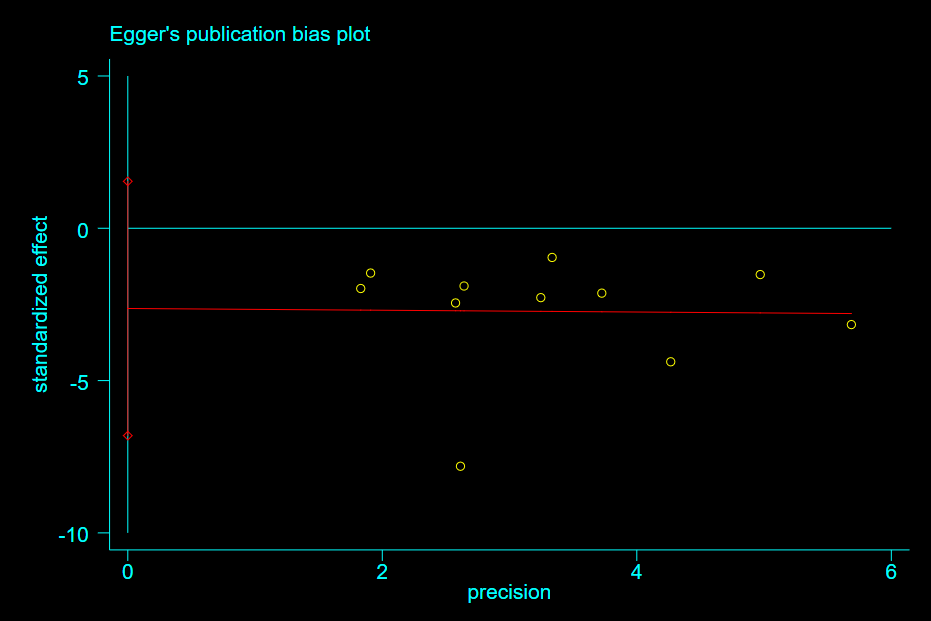


Figure 2 Egger’s publication bias plot for PSQI score. PSQI:Pittsburgh sleep quality index.


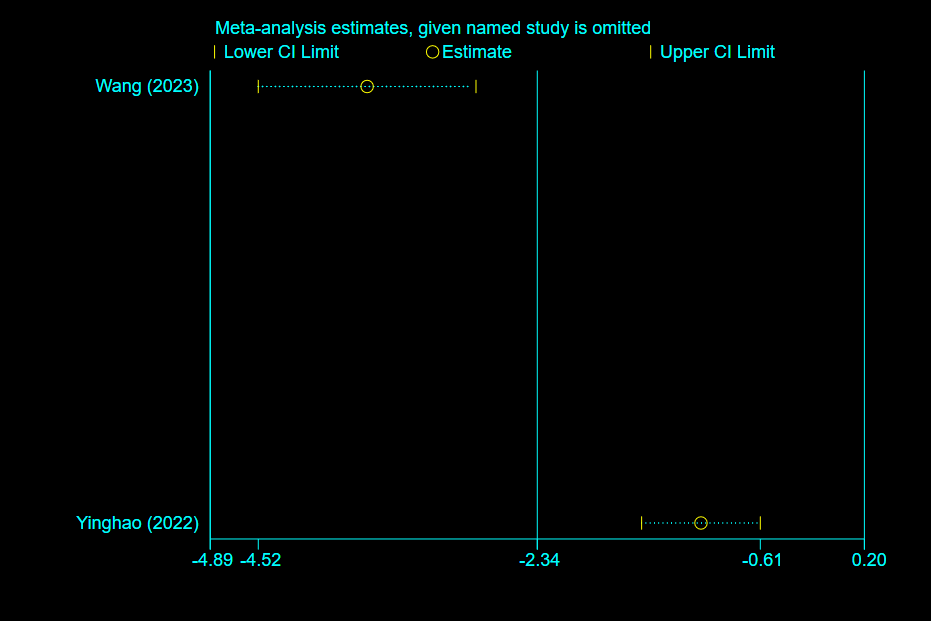


Figure 3 Sensitivity analysis for AIS score. AIS:Athens insomnia scale. CI: confidence interval.


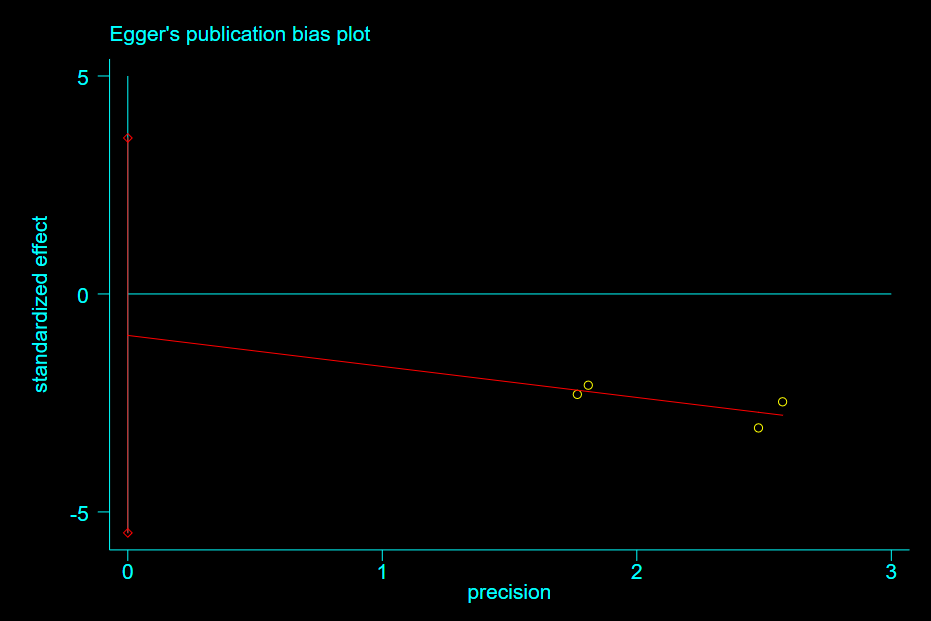


Figure 4 Egger’s publication bias plot for ISI score. ISI:insomnia severity index.


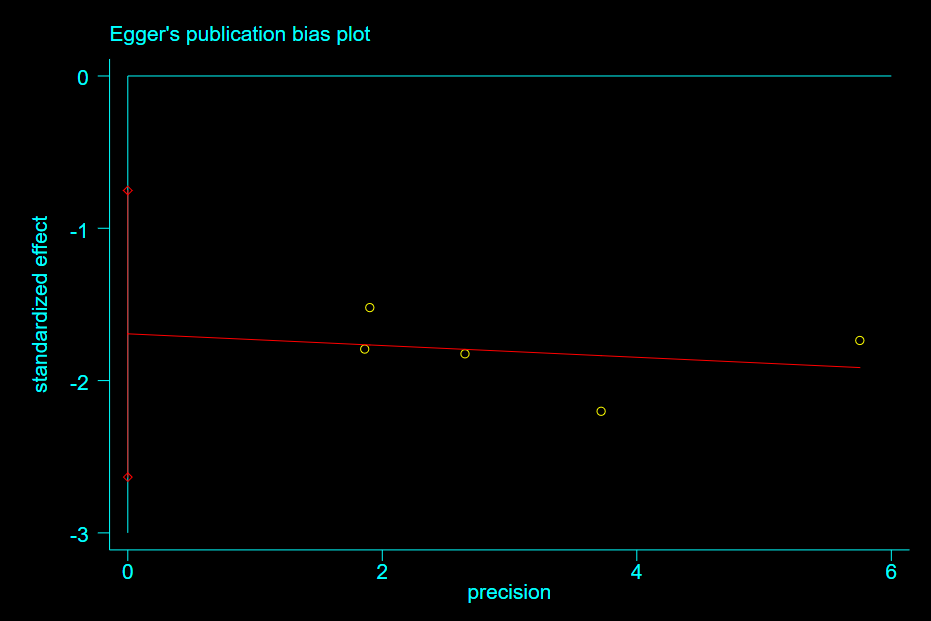


Figure 5 Egger’s publication bias plot for SL score. SL:sleep latency.


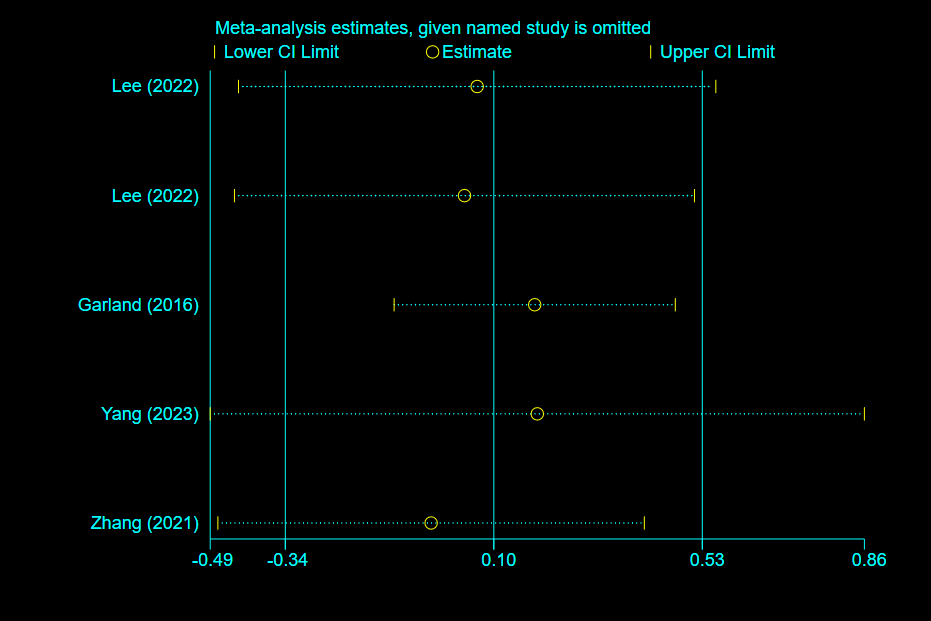


Figure 6 Sensitivity analysis for SE score. SE:sleep efficiency; CI: confidence interval.


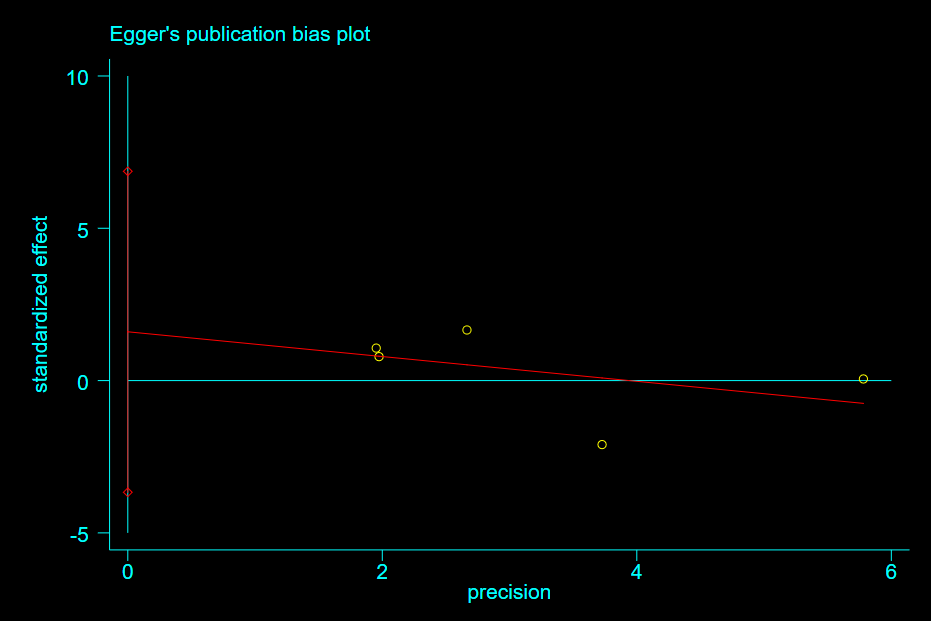


Figure 7 Egger’s publication bias plot for SE score. SE:sleep efficiency.


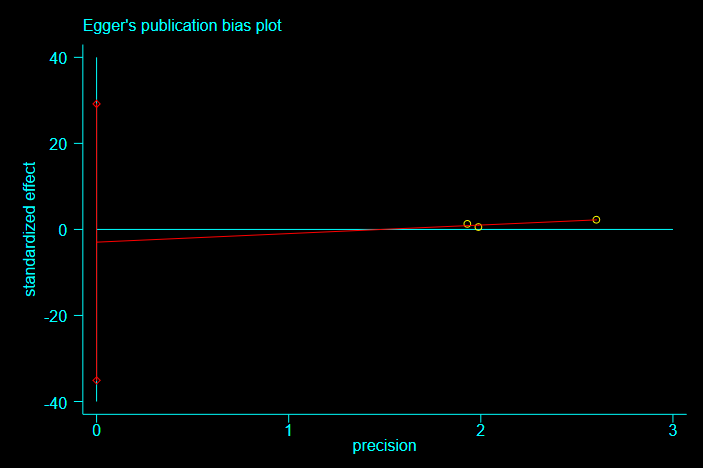


Figure 8 Egger’s publication bias plot for TST score. TST: total sleep time.


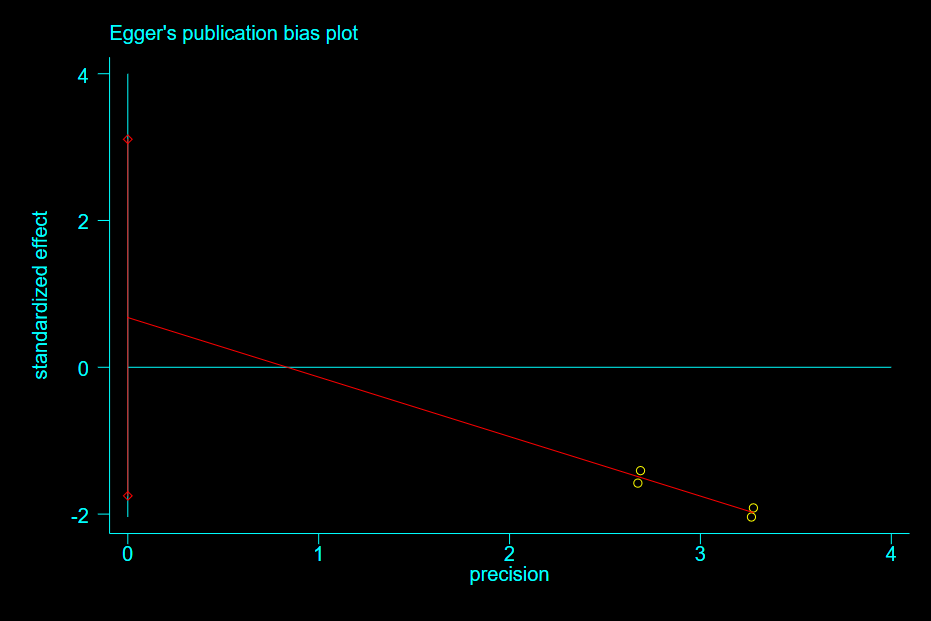


Figure 9 Egger’s publication bias plot for HADS-Anxiety score. HADS: Hospital Anxiety and Depression Scale anxiety.


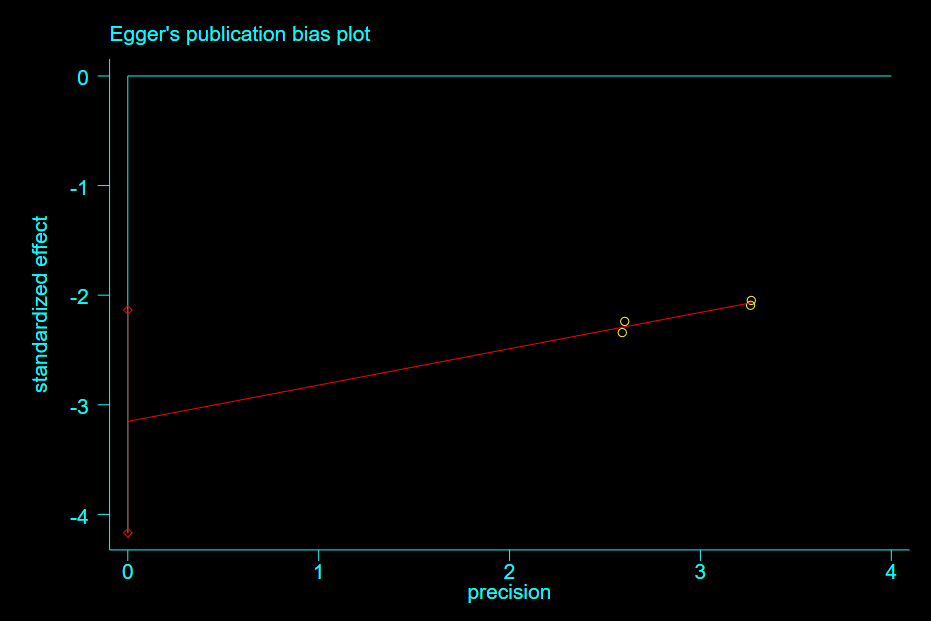


Figure 10 Egger’s publication bias plot for HADS-Depression score. HADS: Hospital Anxiety and Depression Scale depression.

Table 1 GRADE evidence profile for the studies in the meta-analysis

| Outcomes | Grade |
| --- | --- |
| PSQI score | moderate |
| AIS score | moderate |
| ISI score | moderate |
| SL score | moderate |
| SE score | Low |
| TST score | Low |
| SD score | Low |
| SDB score | Low |
| SQ score | moderate |
| HADS-Anxiety score | moderate |
| HADS-Depression score | moderate |
